# Kiwi Plant Canker Diagnosis Using Hyperspectral Signal Processing and Machine Learning: Detecting Symptoms Caused by *Pseudomonas syringae* pv. *actinidiae*

**DOI:** 10.3390/plants11162154

**Published:** 2022-08-19

**Authors:** Mafalda Reis-Pereira, Renan Tosin, Rui Martins, Filipe Neves dos Santos, Fernando Tavares, Mário Cunha

**Affiliations:** 1 Faculdade de Ciências, Universidade do Porto, Rua do Campo Alegre, 4169-007 Porto, Portugal; 2 Institute for Systems and Computer Engineering, Technology and Science (INESC TEC), Campus da Faculdade de Engenharia da Universidade do Porto, Rua Roberto Frias, 4200-465 Porto, Portugal; 3 CIBIO, Centro de Investigação em Biodiversidade e Recursos Genéticos, InBIO Laboratório Associado, Campus de Vairão, Universidade do Porto, 4485-661 Vairão, Portugal; 4 BIOPOLIS Program in Genomics, Biodiversity and Land Planning, CIBIO, Centro de Investigação em Biodiversidade e Recursos Genéticos, Campus de Vairão, Universidade do Porto, 4485-661 Vairão, Portugal

**Keywords:** actinidia, leaf bacterial canker, *Pseudomonas syringae*, plant pathology, in-situ diagnosis, hyperspectral spectroscopy, feature selection, support vector machine

## Abstract

*Pseudomonas syringae* pv. *actinidiae* (Psa) has been responsible for numerous epidemics of bacterial canker of kiwi (BCK), resulting in high losses in kiwi production worldwide. Current diagnostic approaches for this disease usually depend on visible signs of the infection (disease symptoms) to be present. Since these symptoms frequently manifest themselves in the middle to late stages of the infection process, the effectiveness of phytosanitary measures can be compromised. Hyperspectral spectroscopy has the potential to be an effective, non-invasive, rapid, cost-effective, high-throughput approach for improving BCK diagnostics. This study aimed to investigate the potential of hyperspectral UV–VIS reflectance for in-situ, non-destructive discrimination of bacterial canker on kiwi leaves. Spectral reflectance (325–1075 nm) of twenty plants were obtained with a handheld spectroradiometer in two commercial kiwi orchards located in Portugal, for 15 weeks, totaling 504 spectral measurements. Several modeling approaches based on continuous hyperspectral data or specific wavelengths, chosen by different feature selection algorithms, were tested to discriminate BCK on leaves. Spectral separability of asymptomatic and symptomatic leaves was observed in all multi-variate and machine learning models, including the FDA, GLM, PLS, and SVM methods. The combination of a stepwise forward variable selection approach using a support vector machine algorithm with a radial kernel and class weights was selected as the final model. Its overall accuracy was 85%, with a 0.70 kappa score and 0.84 F-measure. These results were coherent with leaves classified as asymptomatic or symptomatic by visual inspection. Overall, the findings herein reported support the implementation of spectral point measurements acquired in situ for crop disease diagnosis.

## 1. Introduction

**Figure 1:**
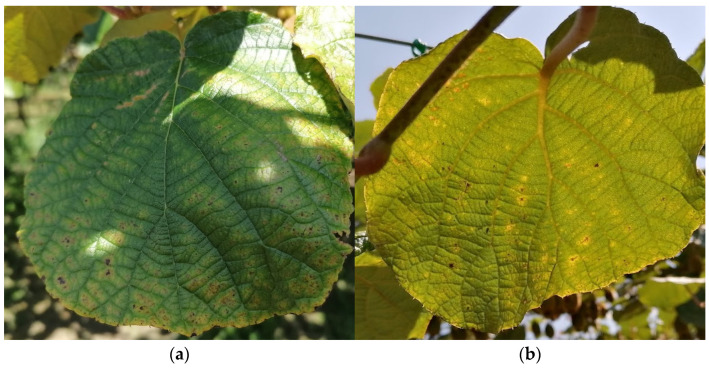
Typical symptoms of Bacterial Canker of Kiwi (BCK) caused by *Pseudomonas syringae* pv. *actinidiae* (Psa) on the adaxial (**a**) and abaxial (**b**) sides of leaves in an advanced stage of the disease.

Bacterial canker of kiwi (BCK) is an emerging disease caused by the Gram-negative bacteria *Pseudomonas syringae* pv. *actinidiae* (Psa), which are responsible for several epidemics and important losses in kiwi production worldwide [1,2,3,4]. In the early stages of the disease, the Psa pathogen colonizes the surface of the host plant without causing significant lesions, but after systemic invasion, may cause severe damage and even death [5,6,7]. Therefore, the early stage of Psa infection may pass unnoticed as the plant has no macroscopic manifestations of the disease (symptoms), jeopardizing the efficiency of phytosanitary procedures to contain the disease [8]. In turn, advanced stages of the infection are more easily detectable since they present characteristic symptoms, consisting of brown leaf spots with chlorotic yellow haloes (Figure 1), necrotic discoloration of buds, cankers with exudate on trunks and twigs, and collapsed fruits [4]. This symptomatologic manifestation reveals that there is a microbial load that has probably already spread to other plants, making it difficult to implement control measures. Thus, it is crucial to develop an early and rapid in situ diagnostic tool for controlling the spread of Psa, through frequent and inexpensive monitoring.

Current diagnostic procedures usually focus on scouting and laboratory-based techniques. The first consists of the inspection of fields (generally visual) by specialized trained observers, to detect and identify infected plants based on the presence of disease symptoms [9]. It is subjective, error-prone (since symptoms alone are not entirely disease-specific), labor-intensive, time-consuming, and expensive [10,11,12,13]. Laboratory-based methods, in turn, include serological and molecular tests and are generally applied due to their sensitivity, accuracy, and effectiveness. The most common laboratory methods include the enzyme-linked immunosorbent assay (ELISA) and polymerase chain reaction (PCR). They entail detailed sampling procedures, which require several hours to be completed, and involve disruptive sample preparation, not allowing a follow-up of the disease progression nor its field mapping to support precision agriculture systems (e.g., site-specific management) [14,15]. Since these laboratory methods were designed to confirm the presence of pathogens, they do not have the necessary high throughput and speed required for supporting real-time agronomic decisions in field extensions. Moreover, they still present some diagnostic limitations, mainly in the asymptomatic and early stages of the disease infection process, due to the uneven spread of pathogens inside plants [14,15]

Innovative plant disease diagnostic tools are expected to provide additional information, namely related to plant–pathogen interactions and resulting changes in the host’s biochemical and biophysical behavior, to that currently generated by the conventional methods mentioned above and should be combined with them. Furthermore, these new techniques, namely spectroscopic approaches, must allow a faster and earlier diagnosis of the disease, and ultimately its field mapping, contributing to more precise agricultural practices. Phytosanitary products can, thus, be applied in the exact area, moment, and dose as required, resulting in a reduction in chemical usage, and consequently in fewer expenses for the producer, residues in crop production, and environmental contamination [16].

Hyperspectral spectroscopy (HS) is a non-invasive and high-throughput technology for measuring early indicators of BCK [17]. HS has been successfully applied in the assessment of a wide variety of plant structural, chemical, biophysical, and metabolic traits in living tissues [18,19,20,21,22]. HS also performed well in the detection of pests [23,24] and phytopathogenic fungi [25,26], bacteria [27], and viruses [28] affecting different crops, even at asymptomatic stages [29]. Through spectral measurements in the visible (VIS, 400–700 nm), and infrared (IR, 800–2500 nm), HS captures quantitative and qualitative changes in the optical properties of plant tissue, which derive from modifications in pigments, sugars, and water levels (among other constituents) [30,31,32,33]. In a simplified way, plants’ spectral behavior in VIS wavelengths is mainly related to pigment concentration and physiological processes (such as photosynthesis). In turn, in the IR region it is mainly correlated with leaf water levels, chemical composition (namely lignin and protein content), structure, and internal scattering processes [34,35]. This information is super-imposed in the recorded spectra at different scales of interference [21,36]. Thus, the detection of BCK using spectral information can be based on the existence of a particular sequence of both metabolic and structural changes, promoted by host–pathogen interactions, which result in the development of characteristic symptoms and, consequently, in modifications in plants’ spectral behavior in VIS–NIR.

HS data may contain a large amount of redundant information from adjacent bands, and only a few wavelength features might be interesting in classifying a diseased plant [37,38,39]. Appropriate strategies usually involving statistical signal-processing approaches, mathematical combinations of different spectral bands, and predictive modeling techniques that can be applied to analyze spectral data and extract useful information and contribute to dimensionality reduction and wavelength selection [32,40,41,42,43,44,45]. Machine learning (ML) algorithms have also been applied to handle the high dimensionality of hyperspectral information [46]. Several modeling approaches have been computed in previous studies to identify and classify plant stress and diseases from spectral data, using either direct spectral reflectance data or information with reduced dimensionality/features selected [47,48,49,50]. The present research aims to explore the suitability and discrimination capability of different multi-variate and machine learning methods in the distinction of asymptomatic and symptomatic kiwi leaves affected by bacterial canker disease, using in-situ, ground-level, UV–VIS hyperspectral measurements. Modeling approaches evaluated the performance of the flexible discriminant analysis (FDA), general linear model (GLM), partial least squares (PLS) classification, and support vector machines (SVM, with different kernels and class weights) algorithms. The data gathered and the proposed workflow are expected to be a robust contribution to extend the HS approaches to plant disease diagnostics in field settings.

## 2. Results

### 2.1. Spectra Filtering and Feature Selection

**Figure 2:**
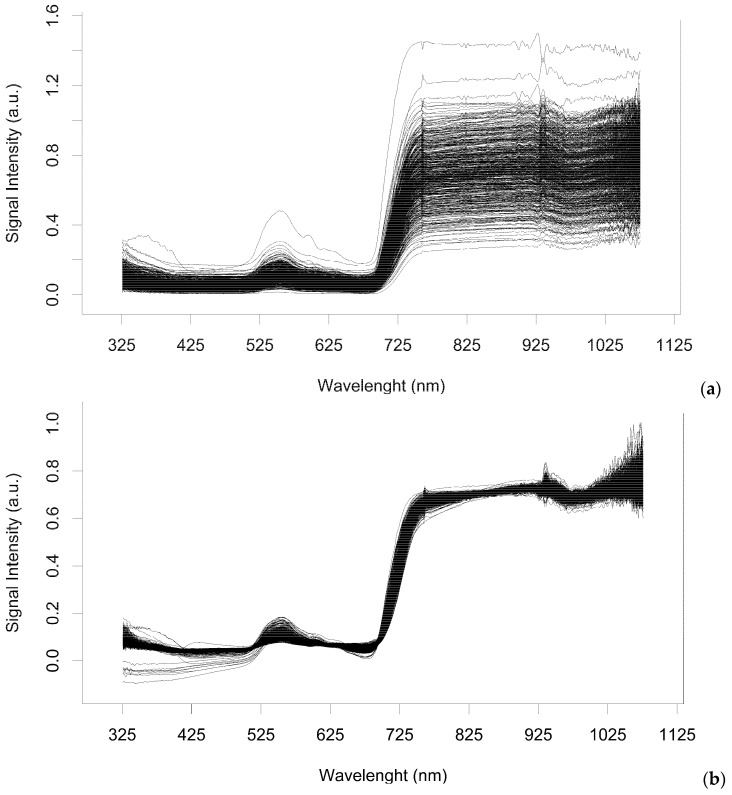
Representation of the spectra collected (**a**), and after its filtering (**b**) using the MSC log algorithm.

After data scatter correction using the MSC log algorithm (Figure 2), an SFFS + JM strategy was computed to assess separability between asymptomatic and symptomatic leaves as a function of the wavelength variables. From a total of 751 predictors in the VIS–NIR spectral region, the procedure selected 33 variables (Table 1) essentially involving wavelengths located in the blue (326–408 nm), green (562, 583 nm), and NIR (777–1068 nm) regions. The JM value was 1.41 indicating high separability between variables.An SFVS approach was also performed for feature choice within the initial 751 predictor candidates. The 35 wavelengths chosen are described in Table 2, including features belonging to the blue (388–446 nm), green (510–556), red (671–754 nm), and NIR (759–1070 nm) regions.

With built-in feature selection, the FDA model only identified seven variables from the total predictors. They belonged to the blue region (424 and 464 nm), green (549 nm), red (719,753 nm), and NIR (759,935 nm) regions. In turn, GLM with the built-in stepwise feature selection sorted out 20 predictors, mainly localized in the blue (388–443 nm), green (510 nm), and NIR (759–1066 nm) regions.

The LASSO method recognized 22 predictors from the total 751 wavelengths available. These spectral features fitted the blue (329–375 nm), green (510, 536 nm), red (617, 671 nm), and NIR (771–1070 nm) regions.

All feature selection methodologies identified similar wavelengths and spectral bands important for discriminating BCK detection.

### 2.2. Model Discrimination of Psa Leaf Symptoms

Table 2 presents the metric values used to compare the model approaches computed to discriminate between asymptomatic and symptomatic kiwi leaves infected by the Psa pathogen, based on random sampling (with no temporal sequence correlated in the samples). Considering all of the available 751 predictors, the mean metrics of the three sets studied (total, BT, and CT data), including all the tested modeling approaches, presented mean values ranging from 0.71 to 0.82 for accuracy, 0.36 to 0.63 (fair to good agreement) for kappa, and 0.65 to 0.81 for the F-measure. In turn, CV ranged from 2.15 to 3.45, 2.62 to 10.16, and 4.57 to 15.18 for the same metrics.

Three independent feature selection methods were then applied and combined with the same models (except for FDA) to verify if selected wavelengths would improve model performance for the discrimination of Psa disease. For the SFVS approach, the mean metric values of the three sets studied ranged from 0.76 to 0.85 for accuracy, 0.49 to 0.69 (moderate to good agreement) for kappa, and 0.71 to 0.83 for the F-measure. The CV scores ranged from 0.07 to 5.37 for accuracy, 2.12 to 12.87 for kappa, and 2.94 to 12.43 for the F-measure. For the SFFS + JM procedure, similar findings were observed, and the mean results covered the interval 0.73 to 0.81 for accuracy, 0.40 to 0.59 (moderate agreement) for kappa, and 0.63 to 0.77 for the F-measure. The CV numbers fluctuated from 0.97 to 4.10, 2.71 to 26.16, and 6.21 to 31.95 for accuracy, kappa, and the F-measure, respectively. These approaches, thus, generally showed higher relative dispersion of the data points in the datasets around the mean, for all the metrics. Lastly, for Lasso, the mean outcomes extended from 0.75 to 0.83, 0.46 to 0.65 (moderate to good agreement), and 0.63 to 0.82 for accuracy, kappa, and the F-measure, respectively. CV, for the same metrics, registered values of 1.78 to 4.48, 7.52 to 12.28, and 2.78 to 21.19.

Between models, the selection was achieved by determining the mean and the CV for the global (encompassing the training and testing data), BT, and CT datasets. The SFVS followed by an SVM algorithm with radial kernel and class weights (stepsvmrw) presented a higher mean (accuracy of 0.85, kappa of 0.69, and an F-measure of 0.83) and lower CV (0.45 for accuracy, 2.12 for kappa and 5.20 for the F-measure) for the different metrics. This model was, hence, selected.

Table 3 presents the confusion matrix for the selected model (stepsvmrw) for the three validation datasets. In the predictions using the total (training and validation set) data, the model correctly classified 190 (TP) spectra of the 223 spectra acquired over the symptomatic leaves (33 observations were wrongly classified—FN). The spectra acquired over the asymptomatic leaves allowed the correct classification of 240 (TN) of the 281 spectra (41 cases of FP) (Table 3).

**Figure 3:**
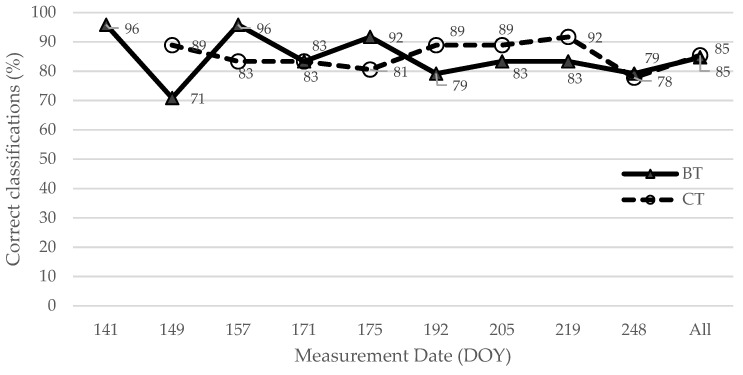
Percentage of correct classification predictions as ‘asymptomatic’ by date and test site using the SFVS strategy, followed by an SVM algorithm with radial kernel and class weights (stepsvmrw model). Values of BT site are represented with triangles and CT with circles. DOY—Day of the year.

Figure 3 presents the temporal prediction trend of correct classification as ‘asymptomatic’ in both test sites, based on the stepsvmrw model. According to dates and test sites, the percentage of cases where the stepsvmrw model attributed the correct classification as ‘asymptomatic’ to each observation ranged from 71% to 96% (Figure 3). The percentage of asymptomatic observations correctly classified decreased for the BT region over time but showed an inverse tendency for the CT site. The BT orchard presented more advanced symptoms of BCK and their growth was relatively stable throughout the measurement period. The lower values of correct asymptomatic class prediction of the last dates can be related to disease asymptomatic leaves showing a spectral signature more similar to symptomatic samples than healthy ones. In turn, for the CT region, spectral measurements allowed complete surveillance from the appearance and development of the first signs of BCK to its full development throughout the time, coinciding with the visual separation between healthy and diseased leaves.

**Figure 4:**
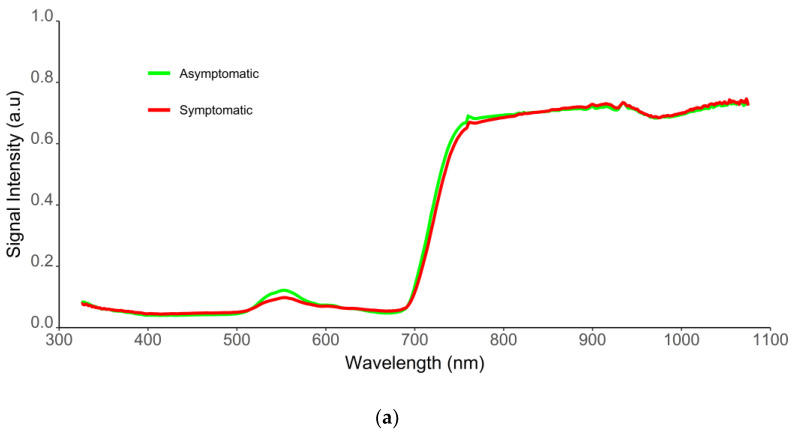

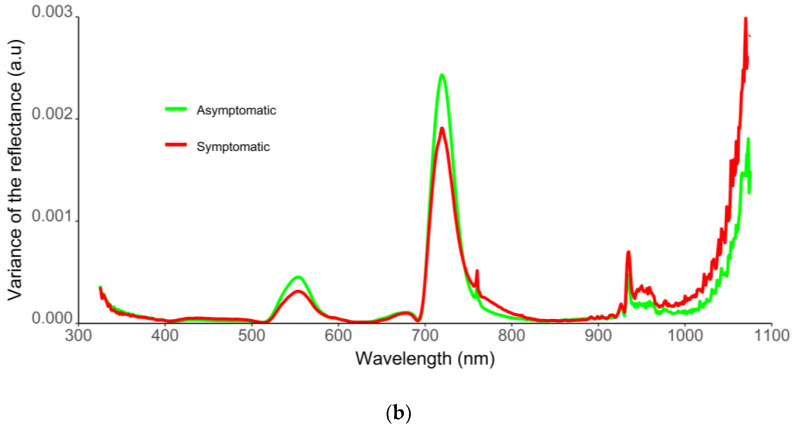
(**a**) Median of the spectra of the 25% observations best classified as ‘asymptomatic’ (green) and ‘symptomatic’ (red) for the selected model combining the SFVS with SVM with radial kernel and class weights (stepsvmrw); (**b**) Variance of the reflectance data measured by spectral wavelength and class (green line representing the variance in the mean spectra of ‘asymptomatic’ samples, and red line illustrating the variance in the mean data of ‘symptomatic’ leaves).

Figure 4a represents the median spectra of the 25% of observations classified with higher probability as ‘asymptomatic’ and ‘symptomatic’ by the predict function of the ‘caret’ package which was computed for the selected model. Reflectance curves of asymptomatic samples were characteristic of healthy green leaves, presenting lower reflectance values in the VIS spectral region, and a high reflectance level in the NIR region. In turn, symptomatic samples showed characteristic, divergent reflectance curves. Visual changes were observed between asymptomatic and symptomatic samples for wavelengths ranging from 515–650 nm (green–yellow–orange region), 651–714 nm (red region), and 715–850 nm (red-edge and NIR regions). Higher reflectance values were observed for the blue region (450–520 nm) and most NIR regions (850–1075 nm) for symptomatic leaves compared to the asymptomatic ones. The opposite tendance was observed in the green, red-edge, and beginning of the NIR region (<850 nm). Nevertheless, spectral variance (Figure 4b) was reduced for wavelengths higher than 800 nm.

## 3. Discussion

Proximal sensing techniques can be a useful tool for helping producers detect early crop diseases in situ. However, qualitative and/or quantitative differences between the spectral information according to leaf symptomatology must be retrieved. In this regard, our study investigated the possibility of using different model approaches of hyperspectral data to correctly classify kiwi leaves according to the presence of characteristic symptoms of BCK disease. The analysis was performed in two kiwi orchards, where 504 spectral signatures were randomly acquired from symptomatic (diseased) and asymptomatic kiwi plant leaves over time (Table 4). Monitoring of these two kiwi orchards allowed the evaluation of the impact of different environmental and meso- and microclimatic conditions, and the influence of different agricultural practices and plant age on model development. A cross-validation strategy was applied to test the null hypothesis, which was assumed to occur when the training and validation sets are randomly sampled, resulting in similar predictions in both datasets. An n-series random sampling can, furthermore, be performed to assure a general evaluation of the error. Hence, cross-validation models can be derived from all datasets, taking the error of a predicted sample [51,52]. Model transferability was later demonstrated by the results obtained in the modeling process.

**Figure 5:**
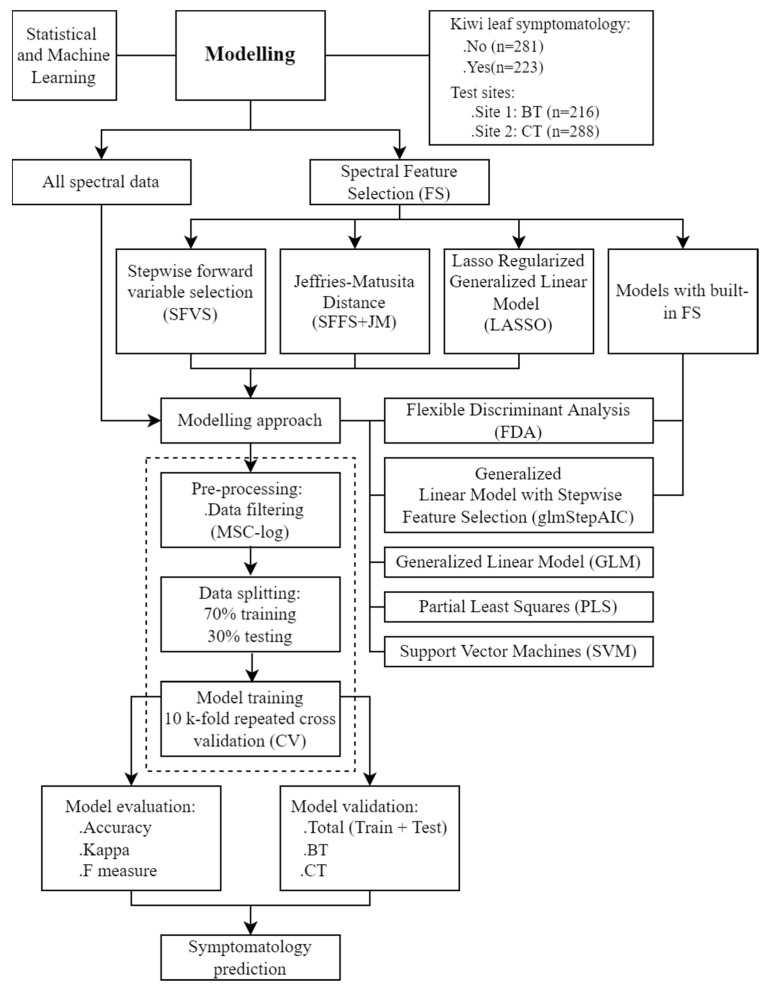
Conceptual diagram for the predictive modeling approaches of bacterial canker of kiwi (BCK).

Hyperspectral data is acknowledged for containing many redundant adjacent features, prone to multicollinearity [53], and suggested feature selection allows the identification of the most relevant information (Figure 2). Hyperspectral data may, in fact, hold limited useful information, reducing model performance due to overfitting, and increasing computational time [28]. Thus, different feature selection techniques were applied to hyperspectral filtered data to identify relevant features having significance in the classification process, namely a sequential forward floating selection using Jeffries–Matusita distance (SFFS + JM), a stepwise forward variable selection method using Wilk’s Lambda criterion (SFVS), and a Lasso regularized generalized linear model (LASSO). Furthermore, two models with built-in feature selection techniques were also computed, specifically the generalized linear model with stepwise feature selection (glmStepAIC) and the flexible discriminant analysis (FDA) (Figure 5).

All approaches (Figure 5) identified similar spectral wavelengths located mainly in the blue (350–500 nm), green (500–600 nm), red (600–750 nm), and NIR (>750 nm) regions (Table 1). These results are coherent, presenting biological significance since the symptoms caused by *Pseudomonas syringae* pv. *actinidiae* (Psa) promote modifications in leaf biochemical and structural composition, as previously mentioned. These selected features for discriminating asymptomatic and symptomatic kiwi leaves are in line with those found for other crops with different diseases, namely: (i) for grapevine, where wavelengths near the green region of the visible (534, 576, 430, and 368 nm), and near-infrared spectra were selected by a stepwise-based approach [54]; (ii) also for grapevine, other wavebands also seem to have high discriminatory power, being mainly located at the green (520–550 nm), chlorophyll-associated wavelengths (650–670 nm), red edge (700–720 nm), beginning of near-infrared (800–900 nm) and shortwave infrared spectral regions [55]; (iii) for soyabean, wavelengths in the green and red regions of the spectrum (top ten wavebands selected by: linear discriminant analysis—523, 535, 592, 658, 694, 700, 733, 766, 931, 1015; logistic discriminant analysis—400, 421, 427, 559, 571, 589, 679, 682, 688, 703; and linear correlation analysis—458, 461, 476, 479, 485, 494, 500, 626, 632, 686) similarly exhibit the best correlation with disease [48]; (iv) for wheat affected by *Puccinia triticina*, the relevant spectral characteristics corresponded to the wavelengths of 605, 695, and 455 nm, for various levels of the infection [56]; (v) for oil palms diseased with ganoderma basal stem rot disease, the features with higher importance were found mainly in the green (from 550 to 560 nm), and in the red-edge (around 650 to 780 nm) regions [44]; (vi) for rice, different levels of panicle blast could be differentiated at six different effective wavelengths, specifically 459, 546, 569, 590, 775, and 981 nm [57].

In crop remote sensing studies, spectral vegetation indices (VIs) are still the most common approaches studied to identify and manage abiotic and biotic stresses in different crops [58,59,60]. VIs are composed of numerous combinations of different bands, providing spectral information with reduced dimensionality [32,61,62]. Despite its extended usage and utility, it is not always clear if this plethora of VIs is sensitive to the variable of interest and, simultaneously, if they respond insensitively to confounding factors, namely variations of other leaf or canopy properties, background soil reflectance, solar illumination, and atmospheric composition, this may induce variability in the spectral properties of surfaces [61]. In turn, feature selection methods may provide more robust and customized spectral information since they can identify the variables that are effective for modeling data class characteristics, reducing the dimensionality of the original feature space by choosing only the best and minimum subset of features [43].

Data modeling was then performed using different statistical and machine learning approaches applied in the complete dataset and the wavelengths identified by the different feature selection approaches (Figure 5). The mean overall accuracy and coefficient of variation of the models allowed the identification of the combination of a stepwise forward variable selection with a support vector machine with radial kernel and class weights (stepsvmrw) as the best modeling approach among those evaluated (Table 2). In this model, the kernel trick reduced dimensions and provided the necessary class separation of non-linear features to the support vectors method e.g., [62]. However, kernels are not theoretically derived for spectroscopy [21]. This handicap may lead to non-optimal selection, that does not represent the relationship between spectral features and discrimination among symptomatic and asymptomatic leaves. This might explain the better performance of SVM models when combined with feature selection algorithms (e.g., stepwise feature selection; SFVS).

Stepsvmrw presented a classification accuracy of 85%, kappa score of 0.70 (good agreement), and f-measure of 0.84, when the total dataset (training and test sets) was used for prediction. It correctly classified 190 spectra of the 223 spectra acquired over the symptomatic leaves and classified 240 of 281 spectra belonging to asymptomatic observations. The percentage of asymptomatic observations correctly classified by this model ranged from 71% to 96% for both test sites, having decreased for the BT region over time but showing an inverse tendency for the CT region (where it increased) (Figure 3). The misclassification regarding the symptomatology of leaves in the early stages (Table 3) may indicate initial disease phases in the NIR domain of the spectrum when typical disease symptoms (e.g., chlorosis and necrosis) are not yet visually detectable by the human eye. In turn, for the CT region, spectral measurements allowed complete surveillance from the appearance and development of the first signs of BCK to its full development over time, coinciding with the visual separation between healthy and disease leaves.

Our results showed lower accuracies than those found by Lu et al. [63] for classifying strawberry leaves infected with *Colletotrichum gloeosporioides* using multitemporal indoor and in-field assessments. Their classification accuracy for indoor measurements varied from 81.6% to 89.7% for discriminant analysis (FDA), 84.2% to 93.1% for stepwise discriminant analysis (SDA), and 84.2% to 87.5 % for k-nearest neighbor (KNN), corresponding the lower value to the classification accuracy for asymptomatic samples and the higher value to the accuracy of healthy plants. KNN misclassified healthy samples as asymptomatic. In-situ evaluations had lower accuracy scores ranging from 54.7% to 75.8% for FDA, 62.5% to 77.3% for SDA, and 15.4% to 90.6% for KNN. These poorer values obtained in in-field assessments were probably related to limitations in the dataset, namely the asymptomatic sample size being larger than the healthy and symptomatic sample, and uncontrolled environmental conditions acknowledged as the most important variations in sunlight during measurements. Zhao et. al. [45] used three dimensionality reduction algorithms and three machine learning models to classify and identify powdery mildew (*Blumeria graminisf.* sp. *tritici*) on wheat under laboratory conditions. When applied to hyperspectral data, SVM achieved a classification accuracy of 88.0%. The best model combined principal component analysis (PCA), for dimensionality reduction, and SVM, having achieved an identification accuracy of 93.3% by cross-validation methods. The authors only assessed 75 picked leaves, with the number of diseased samples (60) being considerably higher than the number of healthy ones. Huang et al. [64] studied the wheat powdery mildew disease using 145 in-situ hyperspectral measurements (90 healthy and 55 diseased samples), different vegetation indices (alone and combined with each other), and three model classifiers. They obtained classification accuracies ranging from 74.5% to 94.8%. Despite our accuracy values being similar or slightly lower than these examples, their scores were generally obtained by performing indoor assessments (made under supervised, controlled conditions), and/or through modelling approaches developed with small datasets, where spectral noise and variability are low. Moreover, most models were only applied to a single test site, with restricted soil, climate conditions, and plant age, not being able to generalize to a practical application.

Model results were further supported by the empirical analysis of the spectral information of BCK disease. Asymptomatic leaves mostly revealed the typical spectral behavior of green and photosynthetically active vegetation (Figure 4a). In turn, spectral responses of symptomatic leaves registered variations in the VIS and NIR regions; having some spectral bands presenting a greater response to the BCK infection (Figure 4a,b). Overall, the mean spectral reflectance records of symptomatic leaves showed higher values of reflectance for the blue and the majority of the NIR regions (850–1075 nm), and lower values for the red-edge and beginning of the NIR regions (<850 nm), when compared to the asymptomatic cases. These results are consistent with the infection caused by Psa, since it results in necrotic leaf spots, which are related to membrane damage and cell death [4]. Modifications in the content of chlorophyll and brown pigments, water, and structural components influence crop spectral behavior in these spectral regions [65,66]. Other studies, performed on different crops, also reported an increase in diseased leaf reflectance in the VIS region (mainly in the green and red ranges of the spectrum), and a decrease in the NIR region, specifically: (i) sugar beet infected with Cercospora, in the VIS region from 550 to 700 nm and the NIR region from 700 nm to 850 nm [41]; (ii) grapevine infected with leaf stripe disease (esca complex) in the green region (520–550 nm), and red region (650 nm) of the spectra [55]; (iii) soybean affected by the soybean cyst nematode (SCN) and sudden death syndrome (SDS) [48].

Our results are thus relevant for detecting and discriminating the bacterial canker disease of kiwi in leaves. Hyperspectral data provides a large amount of information, allowing the screening of samples based on their chemical composition rather than only their size, shape, and visible color (that RGB devices permit). Despite the promising findings supporting this proof-of-concept, this was a single season, in-field analysis (without control over agronomic, environmental, and infectious conditions). Future studies are thus needed, namely by analyzing the same leaf over time, to better understand the plant–pathogen interaction and its impact on host spectral behavior. Furthermore, supplementary laboratory assessments will be highly beneficial and allow more comprehensive knowledge about the disease caused by the Psa pathogen.

## 4. Materials and Methods

### 4.1. Study Area

The monitoring of kiwi plants (*Actinidia deliciosa*) was performed in two test sites, integrated in commercial orchards at Guimarães, Portugal, located in Caldas das Taipas (CT; 41°29′09.8′′ N 8°21′54.3′′ W) and Briteiros (BT; 41°30′53.3′′ N 8°19′20.5′′ W). In CT, where the orchard was 5 years old when the assay was performed (2020), twelve feminine kiwi plants of the variety Bo.Erika^®^ were selected, marked with tape, and divided according to the presence or absence of visual symptoms characteristic of BCK (small greasy dark spots that become brown to black, that are distributed randomly on leaves, Figure 1). The same procedure was performed for the BT test site, whose orchard was 30-years-old, where eight plants of the same variety were selected to integrate the study.

Disease identification was accomplished by a visual assessment of BCK characteristic symptoms on the kiwi leaf’s adaxial and abaxial sides (Figure 1). Samples were classified as asymptomatic (showing no BCK symptoms) or symptomatic (presenting at least one typical BCK chlorotic or necrotic spot). The monitoring of these two sites allowed the evaluation of the impact of different environmental and meso- and microclimatic conditions, as well as the influence of different agricultural practices and plant age.

### 4.2. Spectral Reflectance Acquisition through Ground Measurements

Leaf hyperspectral data were obtained with a portable spectroradiometer (ASD FieldSpec^®^ HandHeld 2, ASD Instruments, Boulder, CO, USA). Reflectance data were recorded in the wavelength range from 325 nm to 1075 nm, with 1 nm of spectral resolution. The spectroradiometer has a full conical field-of-view angle of 25°. During the data acquisition, the sensor was maintained 30 cm above the kiwi leaf, directed vertically downward (nadir view), giving a sampling footprint close to 13.3 cm. The leaf was placed upon a black card to reduce background noise. Prior to the hyperspectral acquisition, an internal dark calibration was performed, followed by a white calibration through a spectralon (white reference panel).

Measurements were acquired in the nadir position, in cloud-free conditions, between 11:00 and 14:00 h (local time), minimizing changes in the solar zenith angle. Weekly hyperspectral data on plant’s reflectance were obtained between May and June 2020, which corresponded to the full development of Psa symptoms in kiwi plant leaves during the growing season. After, biweekly measurements were performed between July and August 2020. Three random leaves were chosen for each plant, and hyperspectral information was collected from one point, totaling 504 measurement points (Table 1). In each spectral measurement, 10 repetitions were performed and later averaged to minimize the noise effect.

The measurements were balanced regarding the test site and symptomatology (asymptomatic or symptomatic). Nearly 43% of the samples were collected in the BT region, presenting 59% of the typical symptoms of BCK. The remaining 57% of observations were collected in the CT region, where only 33% of them showed visual signs of the disease. In fact, differences in disease intensity were observed, with the BT test site being more severely affected by BCK than CT.

A multiplicative scatter correction log (MSC log) was applied in the hyperspectral reflectance according to [21].

### 4.3. Modelling Approaches

#### 4.3.1. Feature Selection

Hyperspectral data are superimposed and result from multi-scale interference, resulting in an auto-correlated signal at various scales [21,36,53]. The state-of-the-art enumerates several techniques useful for reducing the impacts of this high dimensional, redundant information [32]. One approach consists of feature selection techniques applied to identify the most relevant bands and/or range of bands within hyperspectral data associated with the explaining variable. By directly choosing wavelengths, redundant information is removed, retaining only the more relevant discrimination features. If the removal of wavelengths is distributed, information is maintained with minimal loss since the spectrum is auto-correlated [21,36]. In our study, the performance of different modeling approaches in BCK discrimination was assessed when (Figure 5): (i) all the 751 wavelengths predictors were considered (325–1075 nm), (ii) when built-in features selection models were computed, (iii) and, when different wavelength selection methods were applied, namely a sequential forward floating selection using Jeffries–Matusita distance, a stepwise forward variable selection method using Wilk’s Lambda criterion, and a Lasso regularized generalized linear model. The main goal of feature selection was to capture systematic information, ensuring that the model description of data was optimal without under or overfitting.

##### Sequential Forward Floating Selection Search Strategy and the Jeffries–Matusita (SFFS + JM) Distance

A feature selection using the sequential forward floating selection search strategy and the Jeffries–Matusita (SFFS + JM) distance [67] was computed to assess the spectral separability between the distributions of asymptomatic and symptomatic samples. This approach is an extension of the sequential forward selection algorithm. It comprehends a backward step that allows the variables included in the prior steps to be reconsidered, increasing the number of possible combinations evaluated. The Jeffries–Matusita (JM) distance was selected as a separability metric, whose value ranges from zero to two, with values above 1.9 being considered indicators of clear separability [68]. The JM distance among the distributions of the two classes $\omega_{i}$ and $\omega_{j}\;$ can be calculated by Equation (1) [69]:

(1) $$JM_{ij}=\int_{x}{\left[\sqrt{p_{i}(x|\omega_{i})}-\sqrt{p_{j}(x|\omega_{j})}\right]}^{2}dx\;$$

where *p* (x/$\omega_{i}$) and *p* (x/$\omega_{j}$) are the conditional probability density functions for the feature vector x, given the data classes $\omega_{i}$ and $\omega_{j}$, respectively. It can be rewritten according to the Bhattacharyya distance ($B_{ij})$:

(2) $$JM_{ij}=\sqrt{2\left(1-e^{-B_{ij}}\right)}\;$$

In hyperspectral remote sensing data, class distributions are often modeled as Gaussian distributions [69]. Under this hypothesis, the Bhattacharya distance can be mathematically written as Equation (3):

(3) $$B_{ij}=\frac{1}{8}{\left(\mu i-\mu j\right)}^{T}{\left(\frac{\sum i+\sum j}{2}\right)}^{-1}\left(\mu i-\mu j\right)+\frac{1}{2}ln\left[\frac{1}{2}\frac{\left|\sum i+\sum j\right|}{\sqrt{\left|\sum i\right|\left|\sum j\right|}}\right]$$

where  μ*i* and μ*j* represent the vector means of classes *i* and *j*, respectively, and ∑*i* and ∑*j* are the covariance matrices of the same classes.

JM distance was selected since it is an efficient method for class separation distances. The JM performs good feature ranking for two-class comparisons [70], and shows a saturated performance when the separability between the measured classes increases. When the saturation point is achieved, any further feature provided does not increase the separability [69].

##### Stepwise Forward Variable Selection Method Using Wilk’s Lambda Criterion (SFVS)

A stepwise forward variable selection (SFVS) approach was performed for feature selection within the initial 751 predictor candidates. This procedure is based on determining the predictive variables that most contribute to the model improvement in each step, compared to the model in the previous step. The choice is based on Wilk’s Lambda criterion. This statistic measures distance based on scalar transformations of the covariance matrixes between and within groups [71].

##### Lasso Regularized Generalized Linear Models (LASSO)

Lasso regularized generalized linear models (LASSO) was also computed since this is considered an efficient procedure for fitting the entire Lasso regularization path for linear regression models via penalized maximum likelihood [72,73].

Computing models with built-in feature selection were also tested to compare their performance with the algorithms where the search routine for the right predictors is external to the model. These models generally work by pairing the predictor search algorithm with the parameter estimation and are usually optimized with a single objective function (e.g., error rates or likelihood) [74]. Generalized linear model with stepwise feature selection (glmStepAIC) and the flexible discriminant analysis (FDA) were chosen to integrate this study.

#### 4.3.2. Predictive Modeling in Classification Mode

Seven predictive modeling approaches were evaluated to detect the bacterial canker of kiwi disease (Figure 2). The leaf symptomatology was used as a binary variable in the models tested taking the values ‘No’ (asymptomatic) and ‘Yes’ (symptomatic). The algorithms computed included (i) flexible discriminant analysis (FDA); (ii) general linear model (GLM); (iii) partial least squares (PLS) classification; (iv) support vector machines with linear kernel (SVM-L); (v) support vector machines with radial basis function kernel (SVM-R); (vi) linear support vector machines with class weights (SVM-LW); and (vii) radial support vector machines with class weights (SVM-RW).

##### Flexible Discriminant Analysis (FDA)

The FDA was selected since it is a multigroup nonlinear discrimination/classification and pattern-recognition method based on nonparametric regression followed by linear discriminant analysis (LDA). It uses optimal scoring to convert the response variable so that the data are better for linear separation, and multiple adaptive regression projections to generate the discriminant surface. FDA can be applied with standard linear regression, resulting in Fisher’s discriminant vectors [75,76].

##### Generalized Linear Model (GLM)

GLM was chosen as a parametric, statistical approach that consists of an extension of linear models. GLM establishes the relationships between the explanatory factors and the responses through an estimated regression parameter via confidence intervals [77]. It evaluates the temporal variational pattern of signals instead of their absolute magnitude, being robust in many cases, including severe optical signal attenuations due to scattering or poor contact [78].

##### Partial Least Squares (PLS) Classification

PLS was computed as a multivariate statistic since it proved that PLS is a prominent modeling method capable of dealing with several, multicollinear variables, and in cases where the number of explanatory (number of wavelengths) variables is superior to the number of observations [79]. It aims to minimize the sample prediction error, pursuing linear functions of the predictors that explain as much variation in each response as possible. Also, PLS aims to account for variation in the predictors, under the hypothesis that directions in the predictor space, which are well sampled, should offer an improved prediction for new observations when the predictors are highly correlated [80].

##### Support Vector Machines (SVM)

SVMs were used as a set of machine learning methods built on the concept of optimal separating hyperplane [81], and they can be used for regression and classification tasks [82]. They are non-linear classifiers capable of finding the most extensive margin between two classes in feature space [83]. SVMs have several hyperparameters and different kernel types. The SVM methodology intends to reduce the error test and model complexity [83]. The kernel function transforms raw data inputs from the original user space into kernel space through a user-defined feature map. The kernel functions include linear, polynomial, and radial basis functions (RBF) [84,85]. Some SVMs approaches assign different weights to different data points such that SVM learns the decision surface according to the relative importance of the data points in the training set [86].

##### Model Development and Selection

Symptomatology was then used as the response variable in modeling approaches, and the 751 wavelengths were considered predictor candidates. To run the predictive models, the dataset was divided into training data (70% of random observations) and validation data (30% of the remaining observations) [87], following a holdout method [88]. The training and validation datasets integrate the pairs of concurrent measurements of the symptomatology and the corresponding values of the predicting variables (Figure 2).

For model evaluation criteria, a resampling strategy was considered following a repeated cross-validation strategy using a repeated 10-fold cross-validation to estimate accuracy. The dataset was split into 10 parts, trained in 9, and tested on 1. The process was repeated for all combinations of train–test splits. The final model accuracy was then taken as the mean from the number of repeats [87,88]. This strategy allows the execution of verification steps by the model before the final verification is measured on the testing set, decreasing the possibility of overfitting [89,90].

Different metrics were then considered to assess model performance and model selection, namely the confusion matrix (CM), accuracy score, kappa coefficient, and the F1-score (Figure 2).

The CM presented possible categories of predicted values in one dimension and the possible categories for actual values in the other. Correct classifications (when the predicted value was equal to the actual value) felt on the diagonal in the CM. The off-diagonal matrix cells corresponded to the incorrect predictions, where the predicted value diverges from the actual value. The class of interest was positive, while the other was identified as negative. The prediction was then classified as a true positive (TP) when it was correctly classified as the class of interest; true negative (TN) when it was properly categorized as not the class of interest; false positive (FP) when it was incorrectly considered as the class of interest; and, false negative (FN) when it was mistakenly labeled as not the class of interest.

The accuracy can be considered as the number of correctly classified prediction instances divided by the total number of predictions. The accuracy (also known as success rate) can be calculated through the proportion of TP and TN in all evaluated cases with the confusion matrix results. Mathematically, this can be stated as presented in Equation (3) [88]:

(4) $$\mathrm{Accuracy}=\frac{\mathrm{TP}+\mathrm{TN}}{\mathrm{TP}+\mathrm{TN}+\mathrm{FP}+\mathrm{FN}}$$

The kappa statistic, or Cohen’s kappa, corrects the accuracy by accounting for the possibility of an accurate prediction by chance alone [88]. Its value can vary from zero to one. The interpretation of the kappa statistic may be different according to how a model is to be implemented. The value one indicates a perfect agreement between the model’s predictions and the true values, and values lower than one indicate an imperfect agreement. Usually, kappa results can be interpreted as followed: less than 0.20—poor agreement; 0.20 to 0.40—fair agreement; 0.40 to 0.60—moderate agreement; 0.60 to 0.80—good agreement; and 0.80 to 1.00—very good agreement [88]. The Kappa statistic can be calculated through the following formula, Equation (4):

(5) $$k=\frac{\mathrm{Pr}\left(a\right)-\mathrm{Pr}\left(e\right)}{1-\mathrm{Pr}\left(e\right)}$$

where Pr(*a*) represents the proportion of actual agreement and Pr(*e*) refers to the expected agreement between the classifier and the true values, under the hypothesis that they were chosen randomly.

F-measure (F1 score or F-score) was also used as an indicator of model performance that merged precision (proportion of positive cases that are truly positive) and recall (a measure of how complete the results are, which is computed as the number of TP over the total number of positives) into a single number using the harmonic mean, a type of average that is applied for levels of change, as represented mathematically by the formula in Equation (5):

(6) $$F-\mathrm{measure}=\frac{2\times \;\mathrm{precision}\;\times \;\mathrm{recall}}{\mathrm{recall}+\mathrm{precision}}=\frac{2\times \mathrm{TP}}{2\times \mathrm{TP}+\mathrm{FP}+\mathrm{FN}}$$

These metric scores were applied to the between model selection through a prediction process using the (i) total dataset (including training and test set), and (ii) site-independent datasets (BT and CT observations). Between model selection was ultimately achieved through the evaluation of the mean and the coefficient of variation (CV) values for the different model metrics of the global (training and testing data), BT, and CT sets, being selected the model with an overall higher means and lower CV for the accuracy, kappa, and F-measure metrics.

For the best model, the percentage of correct predictions was determined by dividing the number of cases where the model attributes the correct class to the prediction compared to the actual class through the total number of predictions performed. Also, the median of the spectra of the 25% predictions classified with higher probability as ‘asymptomatic’ and ‘symptomatic’ by the best model was computed.

All the computational analyses were performed in the software R [91] with the following packages: ‘AppliedPredictiveModeling’ [92], ‘caret’ [74], ‘e1071′ [93], ‘earth’ [94], ‘ggplot2’ [95], ‘glmnet’ [72], ‘kernlab’ [96], ‘klaR’ [97], ‘MASS’ [98], and ‘mda’ [99].

## 5. Conclusions

This study proposes the diagnostics of bacterial canker of kiwi (BCK) disease caused by *Pseudomonas syringae* pv. *actinidiae* (Psa), on kiwi leaves using hyperspectral in-field measurements. Asymptomatic leaves revealed the typical spectral behavior of green and photosynthetically active vegetation, while symptomatic leaves presented deviations in their spectral signature in the VIS and NIR regions. The different feature selection methods allowed the identification of several wavelengths as more important for BCK discrimination, being mainly located in the blue (350–500 nm), green (500–600 nm), red (600–750 nm), and NIR (>750 nm) regions. Spectral separability between asymptomatic and symptomatic observations were observed in the dataset, and a stepwise forward variable selection approach with an SVM algorithm with a radial kernel and class weights presented the best results in terms of disease discrimination. The model presented an overall accuracy of 0.85, with a 0.70 kappa score and 0.84 F-measure. Our findings allowed a rapid, non-destructive, in situ disease classification, supporting the implementation of spectral point measurements for crop disease discrimination. Nonetheless, more research is necessary to better comprehend the plant–pathogen dynamics and their effects on host spectral behavior. Furthermore, feature selection approaches for disease diagnosis must be further explored to develop more economic, multiband sensors. Multi- and hyperspectral sensors can be coupled on different platforms, forming distinct functioning measurement systems. This results in more precise agronomic practices, such as mapping, monitoring, scouting, and treatment of crop diseases. Handheld sensors, terrestrial (e.g., robots) and aerial platforms (e.g., drones), and satellites can assess plant spectral behavior on different scales, including leaf, single-plant, canopy, plot, and farm levels.

## Figures and Tables

**Table 1:** Selected discriminative wavelengths for model development.

Method	Selected Discriminative Wavelengths (nm)
*SFFS + JM (n = 33)*	326, 327, 329, 330, 335, 336, 352, 359, 360, 364, 365, 408, 562, 583, 762, 777, 778, 779, 786, 828, 897, 908, 923, 995, 1018, 1031, 1038, 1045, 1057, 1059, 1061, 1067, 1068
*SFVS (n = 35)*	388, 401, 406, 414, 415, 419, 443, 446, 510, 515, 556, 671, 724, 754, 759, 781, 794, 807, 969, 970, 981, 983, 1009, 1027, 1031, 1032, 1035, 1045, 1048, 1049, 1050, 1053, 1066, 1068, 1070
*FDA (n = 7)*	424, 464, 549, 716, 753, 759, 935
*glmStepAIC (n = 20)*	388, 414, 415, 419, 443, 510, 759, 794, 970, 981, 982, 1001, 1031, 1035, 1045, 1048, 1049, 1050, 1053, 1066
*LASSO (n = 22)*	329, 369, 375, 510, 531, 536, 617, 671, 771, 772, 778, 903, 932, 959, 969, 970, 1045, 1048, 1050, 1052, 1061, 1070

SFFS + JM sequential forward floating selection using Jeffries–Matusita Distance; SFVS—Stepwise forward variable selection; glmStepAIC—Generalized linear model with stepwise feature selection; LASSO—Lasso regression (glmnet).

**Table 2:** Validation results for models classifying bacterial canker of kiwi (BCK) disease.

Feature Selection	Model	Validation Set							Statistics of Validation Sets							
		Total			BT			CT			Mean			CV		
		Acc	K	F1	Acc	K	F1	Acc	K	F1	Acc	K	F1	Acc	K	F1
*None*	PLS	0.7083	0.4047	0.6589	0.6806	0.3329	0.7356	0.7292	0.3536	0.5412	0.7060	0.3637	0.6452	3.4530	10.1605	15.1756
N = 751	SVM-L	0.8274	0.6444	0.7883	0.8012	0.6154	0.8313	0.8403	0.6167	0.7262	0.8230	0.6255	0.7819	2.4209	2.6188	6.7574
	SVM-LW	0.8115	0.6274	0.8104	0.7917	0.5464	0.8421	0.8264	0.6324	0.7685	0.8099	0.6021	0.8070	2.1494	8.0180	4.5747
	SVM-R	0.7857	0.5628	0.7500	0.7593	0.5015	0.7969	0.8056	0.5435	0.6818	0.7835	0.5359	0.7429	2.9643	5.8482	7.7908
*Built-in*	SVM-RW	0.8056	0.6066	0.7822	0.7778	0.5367	0.8154	0.8264	0.6073	0.7368	0.8033	0.5835	0.7781	3.0356	6.9508	5.0708
N = 7	FDA	0.7698	0.5339	0.7411	0.7546	0.4876	0.7969	0.7812	0.5013	0.6631	0.7685	0.5076	0.7337	1.7364	4.6856	9.1599
N = 20	glmStepAIC	0.8147	0.6243	0.8342	0.7824	0.5471	0.7283	0.8392	0.6318	0.8814	0.8121	0.6011	0.8049	3.5081	7.8006	13.4507
Mean		*0.7890*	*0.5720*	*0.7552*	*0.7609*	*0.5034*	*0.8030*	*0.8015*	*0.5425*	*0.6863*	*0.7866*	*0.5456*	*0.7539*	*2.7431*	*5.9137*	*5.1895*
*SFVS*	GLM	0.7937	0.5806	0.7636	0.7454	0.4754	0.7826	0.8299	0.6121	0.7380	0.7897	0.5560	0.7614	5.3686	12.8742	2.9395
N = 35	PLS	0.7679	0.5249	0.7247	0.7685	0.527	0.7984	0.7674	0.4553	0.6215	0.7679	0.5024	0.7149	0.0717	8.1217	12.4302
	SVM-L	0.7619	0.5115	0.7143	0.7454	0.4942	0.7769	0.7708	0.4649	0.6292	0.7609	0.4902	0.7068	1.3715	4.8054	10.4888
	SVM-R	0.8512	0.6994	0.8344	0.8426	0.6773	0.864	0.8542	0.6667	0.7742	0.8485	0.6811	0.8242	0.8821	2.4494	5.5521
	SVM-LW	0.7897	0.583	0.7854	0.7778	0.5153	0.8322	0.8125	0.595	0.7404	0.7933	0.5644	0.7860	2.2226	7.6132	5.8401
	**SVM-RW**	**0.8532**	**0.7035**	**0.8370**	**0.8472**	**0.6831**	**0.8716**	**0.8542**	**0.6753**	**0.7857**	**0.8515**	**0.6873**	**0.8314**	**0.4446**	**2.1187**	**5.1982**
Mean		*0.8029*	*0.6004*	*0.7766*	*0.7882*	*0.5621*	*0.8210*	*0.8148*	*0.5782*	*0.7148*	*0.8020*	*0.5803*	*0.7708*	*1.6668*	*3.3257*	*6.9143*
*SFFS + JM*	GLM	0.7202	0.4327	0.6831	0.7222	0.4109	0.7778	0.7500	0.4162	0.5955	0.7308	0.4199	0.6855	2.2794	2.7074	13.3009
N = 33	PLS	0.7242	0.4355	0.6729	0.7407	0.4501	0.7926	0.7257	0.3209	0.4968	0.7302	0.4022	0.6541	1.2495	17.5938	22.7478
	SVM-L	0.7222	0.4253	0.6517	0.7593	0.4894	0.8074	0.7153	0.2849	0.4605	0.7323	0.3999	0.6399	3.2317	26.1576	27.1545
	SVM-R	0.7639	0.5117	0.7047	0.7639	0.5184	0.7935	0.8194	0.5618	0.6829	0.7824	0.5306	0.6270	4.0955	5.1256	31.9489
	SVM-LW	0.7381	0.4637	0.6887	0.7639	0.4984	0.8118	0.7188	0.2957	0.4706	0.7403	0.4193	0.6570	3.0567	25.8569	26.2985
	SVM-RW	0.8075	0.6057	0.7707	0.7824	0.5532	0.8127	0.8333	0.6022	0.7176	0.8077	0.5870	0.7670	3.1509	5.0002	6.2135
Mean		*0.7440*	*0.4747*	*0.6419*	*0.7460*	*0.4791*	*0.6453*	*0.7554*	*0.4867*	*0.7993*	*0.7539*	*0.4598*	*0.6718*	*0.9695*	*8.7409*	*17.3572*
*LASSO*	GLM	0.7560	0.5056	0.7248	0.7176	0.4021	0.7732	0.7847	0.4973	0.6517	0.7528	0.4683	0.7166	4.4724	12.2796	8.5361
N = 22	PLS	0.7560	0.5028	0.7172	0.7407	0.4501	0.7926	0.7674	0.437	0.5939	0.7547	0.4633	0.7012	1.7752	7.5177	14.3045
	SVM-L	0.7599	0.5127	0.7269	0.7361	0.4393	0.7897	0.7778	0.4725	0.6279	0.7579	0.4748	0.7148	2.7601	7.7407	11.4114
	SVM-R	0.8353	0.6654	0.8118	0.8009	0.5842	0.8352	0.8611	0.6774	0.7778	0.8324	0.6423	0.8083	3.6282	7.8933	3.5709
	SVM-LW	0.7639	0.523	0.7373	0.7269	0.4217	0.4807	0.7917	0.5213	0.6739	0.7608	0.4887	0.6306	4.2728	11.8692	21.1945
	SVM-RW	0.8373	0.6708	0.8178	0.8009	0.5828	0.8365	0.8646	0.6913	0.7914	0.8343	0.6483	0.8152	3.8307	8.8915	2.7795
Mean		*0.7847*	*0.5634*	*0.7560*	*0.7539*	*0.4800*	*0.7513*	*0.8079*	*0.5495*	*0.6861*	*0.7822*	*0.5310*	*0.7311*	*3.4659*	*8.4093*	*5.3430*

CV—Coefficient of Variation; Acc—Accuracy; F1—F-measure; GLM—Generalized linear model; glmStepAIC—Generalized linear model with stepwise feature selection; FDA—Flexible discriminant analysis; K—Kappa; LASSO—Lasso regression (glmnet); PLS—Partial least squares; SFFS + JM—Sequential forward floating selection using Jeffries–Matusita distance; SFVS—Stepwise forward variable selection; SVM—Support vector machine (L—Linear kernel; LW—Linear kernel with class weights; R—Radial kernel; RW—Radial kernel with class weights).

**Table 3:** Confusion matrix for the selected model characterized by executing SFVS followed by an SVM algorithm with radial kernel and class weights (stepsvmrw) using the BT, CT, and complete dataset.

BT (*n* = 216)				CT (*n* = 288)				ALL (*n* = 504)			
		Actual value				Actual value				Actual value	
		‘No’	‘Yes’			‘No’	‘Yes’			‘No’	‘Yes’
Predicted	‘No’	71	15	Predicted	‘No’	169	19	Predicted	‘No’	240	33
	‘Yes’	18	112		‘Yes’	23	77		‘Yes’	41	190

‘No’ and ‘Yes’ correspond to asymptomatic and symptomatic leaves, respectively.

**Table 4:** Number of observations (leaves and plants) per test site and symptomatology.

Test Site	Sites	Dates	Plants	Asymptomatic Leaves	Symptomatic Leaves	Total Measurements
Briteiros (BT)	1	9	8	89	127	216
Caldas das Taipas (CT)	1	8	12	192	96	288
Total	2	9	20	281	223	504

## Data Availability

Not applicable.
